# XRF techniques to quantify heavy metals in vegetables at low detection limits

**DOI:** 10.1016/j.fochx.2018.100001

**Published:** 2018-12-15

**Authors:** Harris L. Byers, Lindsay J. McHenry, Timothy J. Grundl

**Affiliations:** Department of Geosciences, University of Wisconsin Milwaukee, Milwaukee, WI, USA

**Keywords:** Chemical compounds studied in this article, Lead (PubChem CID: 5352425), Chromium (PubChem CID: 23976), Nickel (PubChem CID: 935), Copper (PubChem CID: 23978), Zinc (PubChem CID: 23994), Arsenic (PubChem CID: 5359596), Yttrium (PubChem CID: 23993), Cadmium (PubChem CID: 23973), Wavelength dispersive X-ray fluorescence, Portable energy dispersive X-ray fluorescence, Vegetable sample preparation, Urban agriculture, Heavy metals, Lead

## Abstract

Sample preparation techniques, measurement routines and custom calibrations were developed and optimized for the quantification of Pb and additional heavy metals in dried and wet consumable vegetable tissues via WD-XRF and/or portable ED-XRF spectroscopy. After mitigating matrix influences fundamental to XRF quantification of heavy metals in a carbon matrix, the custom measurement and calibration routines were capable of detecting and quantifying Pb in dried prepared vegetable samples at concentrations relevant to the World Health Organization. The viability of each calibration was evaluated with measurements of vegetables grown in metals-rich garden soil collected from residential properties. Although robust calibrations for dried vegetables were obtained with WD-XRF, the slight compromise in the precision and accuracy of measurements with portable ED-XRF is offset by the portability of this technology. Heavy metal concentrations in wet coarsely-homogenized raw consumable vegetable tissues were quantified; however, further work is needed to test the viability of ED-XRF calibrations.

## Highlights

• Calibration routines for quantification of Pb in vegetables with XRF are optimized.
• These XRF methods achieved detection limits of 0.3 μg g^−1^ for Pb in dry vegetables.
• Pb in raw vegetables is quantified at levels as low as 1 μg g^−1^ with ED-XRF.

## 1 Introduction

For lithologic media, the quantification of elements using X-ray Fluorescence (XRF) spectroscopy is well understood and methods of sample preparation, measurement methods, and calibration/quantification are well documented (e.g. Byers, McHenry, & Grundl, 2016). In quantifying element concentrations using XRF, photons of energy are generated by an X-ray source (such as a compact Rh X-ray tube) and the photons pass through one or more primary filters to reduce the variability in source X-ray photon energy. Source photons then pass into a sample and transfer their energy to an inner-shell electron of an atom within the sample, which slightly displaces the electron from its preferred orbit leaving an unstable atom. An electron from an outer orbital then fills the vacancy in the lower orbital and at the same time releases energy in the form of a fluoresced secondary X-ray unique to the element and unique to the energy difference between orbitals. Element concentrations are determined by the rate at which fluoresced secondary X-rays are measured by a detector in the spectrometer.

Due to the non-destructive nature of the analysis, XRF is emerging as a promising method for rapid quantification of heavy metals in vegetables as recent work demonstrates that lead and other heavy metals are taken up and translocated from the soil into consumable vegetable tissues (Clark et al., 2006, Ferri et al., 2015, Finster et al., 2004, Jolly et al., 2013, Lima et al., 2009, Nabulo et al., 2011, Rodriguez-Iruretagoiena et al., 2015, Sekara et al., 2005). Although human exposure and the resulting health impacts from direct contact with lead-contaminated soil is considered to be a primary pathway for lead exposure in urban agriculture, secondary exposure from chronic consumption of vegetables containing lead could be a significant contributing factor in total lead exposure (Chopra and Pathak, 2015, Ferri et al., 2015, Jolly et al., 2013), especially in high-risk populations, such as low-income, immigrant communities where exposure to lead remains disproportionately high. Child exposure to lead leads to a multitude of poor health outcomes (Keller et al., 2017, Lane et al., 2008, Schnur and John, 2014); therefore, further evaluation of lead (and other heavy metals) exposure is paramount.

Established methods for measuring elements in vegetable and herb tissues includes a combination of traditional wet chemistry methods such as atomic absorption spectrometry (AAS) (Bozym et al., 2015, Chopra and Pathak, 2015, Lima et al., 2009, Sekara et al., 2005, Song et al., 2012, Yadav et al., 2015), inductively coupled plasma-atomic emission spectrometry (ICP-AES) or inductively coupled plasma-mass spectrometry (ICP-MS) (Bešter et al., 2013, Finster et al., 2004, Murray et al., 2011, Nabulo et al., 2011, Rodriguez-Iruretagoiena et al., 2015, Wiseman et al., 2013), wavelength dispersive XRF (WD-XRF) (Andersen et al., 2013, Figueiredo et al., 2016), bench-mounted energy-dispersive XRF (ED-XRF) (Anjos et al., 2002, Gallardo et al., 2016, Jolly et al., 2013), and portable ED-XRF (Ferri et al., 2015, Gutiérrez-Ginés et al., 2013, Sacristan et al., 2016, Towett et al., 2016). Recent work quantified heavy metals in algae with portable ED-XRF using a fundamental parameter factory calibration for plastics (Bull et al., 2017, Turner et al., 2017).

Sample preparation when using AAS or ICP-MS to quantify heavy metals in plants involves ashing plant material in a furnace or with concentrated hydrogen peroxide followed by digestion with acid (HNO3, HCLO4, H_2_SO_4_) and/or microwave extraction using one or more concentrated acids. These sample preparation techniques are inherently dangerous and generate a significant hazardous waste stream. Comparatively, XRF sample preparation techniques preserve the sample matrix and minimize waste generation. As noted by many in the literature (e.g. Gallardo et al., 2016), XRF is a promising technique in quantification of lead and other heavy metals in vegetable tissues. However, prior reviews of this technology have identified several limiting factors (Marguí et al., 2009, Palmer et al., 2009, Singh et al., 2017). The greatest obstacles identified in prior studies using XRF to measure heavy metals are achieving a limit of detection within the range of regulatory thresholds and generating consistent results that can be confirmed with another quantification technology. Due to limited commercial availability of reference materials and treatment of XRF spectrometers as “black box”, prior studies involving XRF have largely not controlled for matrix effects by: not matching the reference material matrix to sample matrix; using a multitude of sample preparation techniques; or using XRF standard factory calibrations optimized for non-carbon matrices. Further complicating prior XRF work is the quantitation of heavy metals based on the intensities of wavelengths with known peak overlaps. By addressing these inconsistencies and mitigating matrix effects, we hypothesize that WD-XRF and portable ED-XRF can be used to accurately and rapidly quantify heavy metals in vegetable samples with limits of detection applicable to health-based regulatory thresholds.

Although the use of XRF in quantifying heavy metals in plant matrices has been reported previously, this work describes methods to systematically mitigate matrix effects through development of custom reference materials, building matrix-specific measurement and calibration routines, and confirming the efficacy of the XRF methods by comparison to ICP-MS analysis. Remarkably, no consumption standards exist in the United States for heavy metals in produce or cereals; therefore, this work relies on World Health Organization (WHO) food standards (WHO, 2018). On a broader scale, food security threats are often identified in retrospective studies limited to select food groups, select manufacturers/country of origin, and/or elements; however, with multiple new threats to food security identified each year, use of WD-XRF and portable ED-XRF spectrometry is a promising quality check that could be used at ports of entry by regulators, researchers, and/or growers/manufacturers to identify security risks prior to consumption/exposure.

## 2 Experimental

The single greatest source of bias in XRF measurements of vegetables is inter-element effects due to secondary absorption/enhancement of target wavelengths. Secondary absorption occurs when a fluoresced characteristic X-ray is absorbed by another atom in the matrix rather than returning to the detector, and if the absorbed energy is great enough, the atom will generate additional x-rays characteristic of the atom (direct secondary enhancement). Additional characteristic X-rays generated following secondary absorption can either return to the detector or be absorbed by additional atoms in the matrix and further generate characteristic x-rays (tertiary enhancement). Therefore, absorption/enhancement of characteristic X-rays can significantly alter the rate at which characteristic x-rays return to the detector such that element concentrations in the sample are not represented by the rate of characteristic x-rays. Mitigation of inter-element (matrix) effects therefore is central to all aspects of this experimental design.

The commutability of reference materials to samples is critical in minimizing measurement uncertainty (Byers et al., 2016) and mitigating matrix effects (absorption/enhancement), but has often been overlooked in prior food studies. Commutability is especially critical when using XRF to quantify heavy metal concentrations in homogenized dry and undried (raw) vegetables as the mass attenuation coefficient of the vegetable matrix is very small compared to more common and readily available silicate-based soil reference materials, which were often used in previous food studies using XRF spectroscopy. Unfortunately, metals-rich plant-based reference materials are either no longer commercially available (e.g. NIST SRM 1515, NIST SRM 1547, BCR-60, BCR-100, BCR-279) or represent a very limited continuum of metal concentrations (e.g. NIST SRM 1570a, NIST SRM 1575a, BCR-129, BCR-414, BCR-482, BCR-670, ERM-CD281, IRMM-804).

Because fully developed and verified metals-rich plant-based reference materials are not readily available, custom dried plant-based reference materials were prepared from easily obtainable commercial materials using methods similar to Figueiredo et al. (2016). Sample preparation techniques were developed for quantitation via WD-XRF and portable ED-XRF spectroscopy using the custom dried plant-based reference materials. Lastly, calibration routines were developed and confirmed with paired ICP-MS measurements of the reference materials. Paired XRF and ICP-MS measurements were taken for vegetables grown in garden soil collected from residential properties in the City of Milwaukee, Wisconsin to confirm the viability of the XRF measurement and calibration routines.

Similar wet plant-based reference materials were prepared as an analogue to raw vegetables in an effort to use portable ED-XRF for quantification of metals in the field. Sample preparation techniques for undried (raw) samples and calibration routines were established. Paired WD-XRF and portable ED-XRF measurements were obtained from vegetables grown in garden soil collected from residential properties in the City of Milwaukee to evaluate the viability of the wet plant-based portable ED-XRF measurement and calibration routines.

### 2.1 Dried plant-based XRF measurement and calibration routines

#### 2.1.1 Preparation of dry plant-based reference materials

Twenty-one plastic jars containing 14 g of freeze-dried parsley were purchased from a retail source in Milwaukee, Wisconsin. The parsley was mixed in bulk and dried in an oven at 60C for 48 h. The powdered parsley was powdered by hand and a 30 g (±1 mg) aliquot of parsley power was added to a rotovap flask containing 200 ml of 18 μmho e-pure water and a pre-determined quantity of liquid ICP metals standard containing 100 mg L^−1^ of Al, As, Cd, Cr, Co, Cu, Fe, Pb, Mg, Mn, Ni, K, Na, Zn and 600 mg L^−1^ Y (Instrument Check Standard 7, SpecCerti Prep ®; Metuchen, NJ). The flask was swirled gently to hydrate the parsley and attached to a water-cooled rotovap (Heidolph Schwabach, Germany) operated at 80C and 80 RPM under vacuum to hydrate the parsley with the metals-rich solution while removing latent water. The rotovap process continued for 2 h or until the mixture was the consistency of a thick paste. The parsley was removed from the rotovap, dried in an oven at 60C for 48 h. Dried material was milled in a tungsten carbide shatterbox for 30 s to create a uniform homogeneous dry powder similar to Marguí et al. (2009). The process was repeated in a step-wise fashion using varying volumes of metals standard to create a library of plant-based reference materials with nominal heavy metal (Al, As, Cd, Cr, Co, Cu, Fe, Pb, Mg, Mn, Ni, K, Na, and Zn) concentrations ranging from 0.5 μg g^−1^ to 100 μg g^−1^ dry weight and nominal Y concentrations ranging from 1 to 600 μg g^−1^. A blank reference was created using the same process, but omitting the addition of the ICP Standard.

A 1 g aliquot of each powdered reference material was digested by TestAmerica Laboratories, Inc. (Chicago, Illinois) using concentrated HNO_3_, HCl, and H_2_O_2_ per Method SW 846 3050B (USEPA, 1996). Heavy metal concentrations in the digestions were measured in triplicate at the UWM School of Freshwater Sciences using a high resolution ICP-MS (Thermo Scientific Element 2) to confirm element concentrations in the reference materials. ICP-MS detection limits for heavy metals of concern are less than 0.1 μg g^−1^.

#### 2.1.2 Preparation of dry pressed pellets for XRF analysis

Preparation of typical powdered soil and rock samples for XRF analysis involves either fusing samples with a flux (such as lithium tetraborate) at high temperatures or pressing powdered samples with a carbon-based binding agent under high pressure to create uniform pellets (Byers et al., 2016). Fusion or ashing is impractical; therefore, uniform pellets were created by pressing dried powdered plant samples at 25 t for 60 s in a 40 mm diameter hydraulic die press.

The variability in pellet thickness across the urban agriculture literature is large and is likely a source of bias. Because matrix attenuation is minimal in vegetable samples, source X-rays entering a vegetable sample can pass entirely through the sample and generate fluoresced X-rays from the entire sample thickness. Therefore, the depth of measurement (also referred to as escape depth) for each element wavelength becomes limited by the attenuation of the fluoresced secondary X-rays by the sample matrix, not by the attenuation of the source X-ray. For a given element wavelength, if the sample is thicker than the measurement depth, the net intensity of fluoresced X-rays is independent of sample thickness, and the pellet is considered “infinitely thick.” If the sample is thinner than the measurement depth for a given element wavelength, the pellet is considered “infinitely thin.” If a pellet is infinitely thin with respect to a given element wavelength, the net intensity of fluoresced X-rays is a function of sample thickness (mass) and subject to significant bias between samples unless samples are of uniform thickness. Additionally, vegetable samples considered infinitely thin could be subject to significant bias from characteristic fluoresced X-rays generated by the spectrometer shielding/housing passing backwards through the sample matrix and potentially generating secondary or tertiary enhancement of characteristic X-rays from matrix elements or by passing through the matrix and being directly measured by the spectrometer detector.

**Fig. 1:**
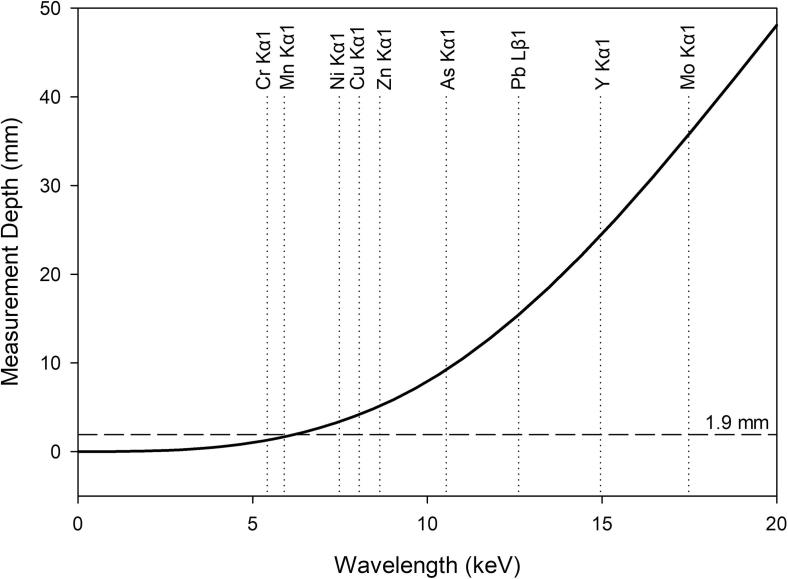
Calculated XRF measurement depth curve for a pressed pellet consisting of Cellulose + Sugar (C_6_H_10_O_5_C_6_H_12_O_6_). Element wavelengths of interest are identified for reference. For a given pellet thickness, wavelengths to the left of the solid curved black line are considered infinitely thick, while wavelengths to the right are not. Pellets used in this study were 1.9 mm thick, which is indicated with a horizontal dashed line for reference.

The depth of measurement (or escape depth of fluoresced X-rays) is calculated based on the matrix density and mass attenuation coefficient for varying X-ray wavelengths of interest (Towett et al., 2016). Unfortunately, the mass attenuation coefficient for plant tissues is unknown and the density of dried vegetables is variable between tissues with reported values ranging from 1.1 g cm^−3^ to 1.7 g cm^−3^ (Martynenko, 2014, Rodríguez-Ramírez et al., 2012). We used a nominal density of 1.4 g cm^−3^ to represent a generic dried vegetable tissue and the mass attenuation coefficient of simple sugar + cellulose (C_6_H_12_O_6_C_6_H_10_O_5_) reported by the National Institute of Standards and Technology (Chantler et al., 2001) for varying wavelengths from 0 to 40 keV (Fig. 1). With these conservative model factors, calculations indicate a dried plant-based sample 1.7 cm thick should be infinitely thick with respect to the Pb Lβ1 wavelength (12.614 keV). Based on the diameter of the pellet die used in this study, a 1.7 cm thick pellet would require approximately 30 g of dried plant material, which is too much powdered material for the XRF pellet die to process. Further, as raw vegetables used in our larger study were 87% water on average, roughly 250 g of raw vegetable would be needed to create a single pellet; which is impractical. Knowing Cr is present inside the housing and shielding of the Pioneer S4 WD-XRF used in this study (Bruker AXS, Inc.), pellets had to be infinitely thick with respect to the CrKα1 wavelength (5.415 keV) to prevent bias in the measurements. To be conservative, 3.2 g of powdered sample per pellet was used and when pressed at 25 t for 60 s, resulted in a pellet approximately 1.9 mm thick. Pellets prepared in this manner are competent, resilient to handling, maintain integrity when stored long-term in a desiccator, and can be analyzed by WD-XRF spectrometry under vacuum without breakage.

**Fig. 2:**
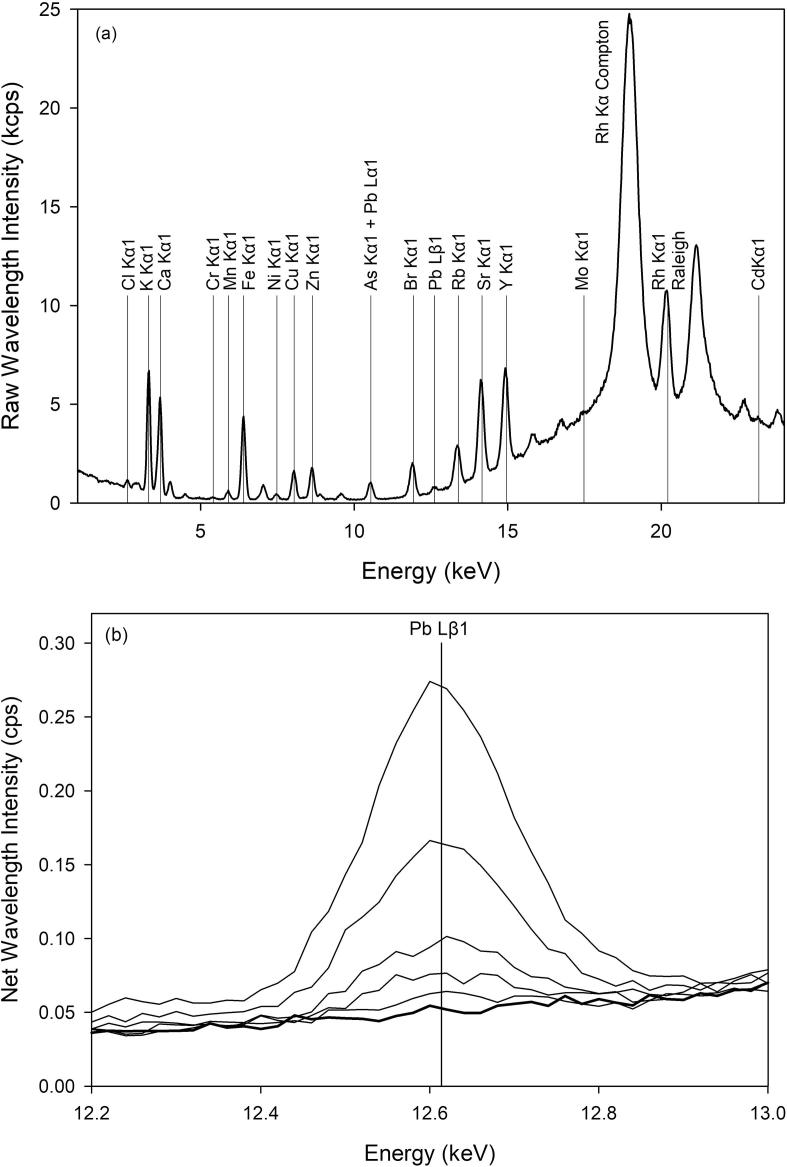
(a) ED-XRF spectrum with the Cu/Ti/Al primary filter of the 10 μg g^−1^ pressed pellet reference material with element wavelengths and Compton/Raleigh peaks identified. (b) the ED-XRF spectra of the Pb Lβ1 wavelength for pellet reference materials with nominal lead concentrations of 0 (heavy line), 5, 10, 20, 50, and 100 μg g^−1^ (successively higher peaks).

Selecting appropriate wavelengths for quantification of heavy metals with XRF is critical to minimize bias and error in measurement routines. Lighter weight elements are commonly quantified based on Kα wavelengths, and due to the keV limitations of the Rh X-ray tube, elements heavier than La are commonly quantified based on Lα wavelengths. However, as shown in Fig. 2a, the Pb Lα1 wavelength overlaps the As Kα1 wavelength. Therefore, to mitigate the peak overlap with As, we used the Lβ1 wavelength for quantification of Pb.

The potential for absorption and enhancement in the matrix was evaluated for each element of interest using data provided by Hubbell and Seltzer (2004). Enhancement of the Pb Lβ1 wavelength is not expected from elements in the parsley matrix, from elements contained in the ICP Standard, or from elements in the shielding/housing. However, as illustrated in Fig. S1 of the Supplemental materials, absorption and secondary or tertiary enhancement of Kα1 wavelengths of Zn, Ni, Fe, Cr, Cu, Co, Mn, and As is possible from elements in the parsley matrix, elements contained in the ICP Standard, and/or elements in the spectrometer shielding/housing.

Using multiple metal foils, we assessed the measurement depth of each element wavelength of interest and possible matrix effects as described in Section 2.1.4 to confirm we had adequately controlled for possible bias associated with pellet thickness and matrix effects.

#### 2.1.3 Development of a dried plant-based WD-XRF measurement and calibration routine

A custom measurement routine was developed in the Bruker AXS, Inc. Pioneer S4 WD-XRF Spectra Plus software to measure the intensities under vacuum of lead using the Pb Lβ1 wavelength and Cr, Ni, and Y using Kα1 wavelengths. Specific analytical details for each element are provided in Table S1 of the Supplemental materials.

Each pellet in the reference material library was analyzed with the WD-XRF measurement routine. Custom calibration routines for each element were developed by comparing net X-ray intensities to known dry weight element concentrations based on element concentrations in each reference material (including the “blank”) as determined by ICP-MS. The routine corrected for rhodium Rayleigh and Compton peaks, matrix effects, and possible contamination of tungsten and cobalt from the shatterbox. Reference materials with element concentrations less than the limit of detection determined by the Pioneer S4 software were omitted from the calibration routines. The goodness of fit, root mean square error, calibration range, the LOD determined by the Pioneer S4 software, and the LOQ calculated per (Thomsen, Schatzlein, & Mercuro, 2003) are listed in Table 1. The coefficient of determination (r^2^) of each calibration is no less than 0.999 with corresponding single-digit root mean square errors.

**Table 1:** Goodness of fit parameters for WD-XRF calibrations of pressed pellets for Cr, Ni, Pb, and Y. The Root Mean Square Error (RMSE) of the calibration, range in reference materials, Limit of Detection (LOD), and Limit of Quantitation (LOQ) are expressed in μg g^−1^, dry weight. The Kα1 wavelengths were used for each calibration, except for Pb which used the Lβ1 wavelength.

	Calibration r^2^	RMSE (μg g^−1^)	Range in Reference Materials (μg g^−1^)		LOD (μg g^−1^)	LOQ (μg g^−1^)
Cr	0.99	1.2	3.1	105	0.6	2.0
Ni	0.99	0.9	6.6	111	0.4	1.3
Pb	0.99	0.7	0.1	96	0.3	1.0
Y	0.99	1.0	0.5	600	0.3	1.0

#### 2.1.4 Confirmation of the dry plant-based WD-XRF measurement and calibration routine

Common garden vegetables were grown in pots of metals-rich soil sourced from residential vegetable gardens in the City of Milwaukee, Wisconsin. The vegetables were harvested, scrubbed vigorously under running water, peeled (root vegetables only), chopped, dried in an oven at 60C for 48 h, milled in a tungsten shatterbox for 30 s, and a 3.20 g aliquot of each sample was pressed at 25 t for 60 s in a 40 mm diameter pellet hydraulic die press. Each sample was analyzed by WD-XRF using the routine described previously and concentrations determined from calibration curves.

Infinite thickness calculations were confirmed using the vegetable pellets by comparing the change in net WD-XRF intensities measurements with and without the presence of a metal foil placed behind dried pressed vegetable samples. The four separate metal foils used in this evaluation include two 99% pure foils of (1) 0.2 mm Cu and (2) 1 mm Pb and two alloy foils of (3) 0.3 mm “nickel-silver” (consisting of 65% Cu, 18% Ni, and 17% Zn) and (4) 0.4 mm stainless steel foil containing Cr and Mn.

**Fig. 3:**
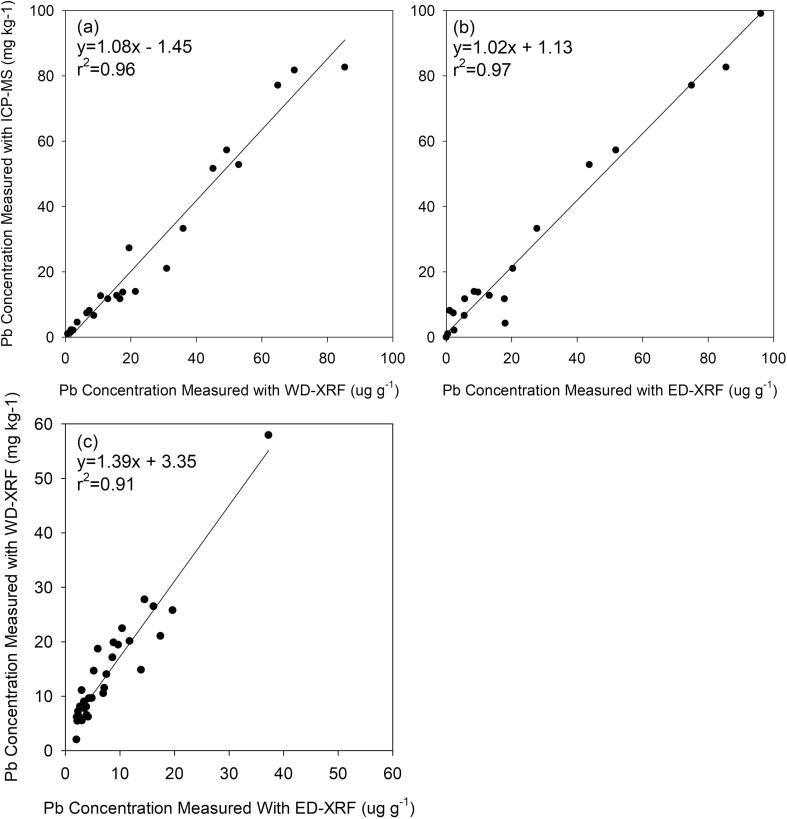
Paired confirmation lead measurements of dried vegetable samples using (a) WD-XRF and ICP-MS, (b) ED-XRF (with the Cu/Ti/Al primary filter) and ICP-MS, and (c) ED-XRF (with the Ti/Fe/Mo primary filter) and WD-XRF. The regression equation for each line and the coefficient of determination (r^2^) are provided.

After measurement with WD-XRF, 23 vegetable pressed pellets representing the range of measured lead concentrations were broken into 1 g aliquots and digested by TestAmerica Laboratories, Inc. using Method SW 846 3050B (USEPA, 1996). Heavy metal concentrations in the digestions were measured in triplicate at the UWM School of Freshwater Sciences using a high resolution ICP-MS (Thermo Scientific Element 2) and concentrations converted to dry weight for comparison between the two analytical techniques. The paired relationships between ICP-MS and WD-XRF measurements of Pb in vegetables are illustrated on Fig. 3a.

#### 2.1.5 Development of a dried plant-based portable ED-XRF measurement and calibration routine

The WD-XRF used in this study is a bench-mounted spectrometer, which limits the usefulness of this technology in real-time quantification of heavy metals in remote locations, such as agricultural fields, gardens, or ports of entry. Portable hand-held ED-XRF is a promising technology for rapid quantification of elements and shows particular promise in the areas of food security/food quality. However, older ED-XRF spectrometers often measured intensities of elements from peaks with known overlaps and relied on default factory calibrations. Recently developed instrumentation allows the entire X-ray spectrum to be captured, stored, and custom calibrations developed based on distinctive element wavelengths. Methods for developing and confirming measurement and calibration routines for quantification of heavy metals in dried pressed pellets with portable ED-XRF are identical to the methods described previously in developing and confirming routines for WD-XRF. In addition to confirming the infinite thickness calculations using metals foils, the influence of measurement time on accuracy and the influence of multi-metal primary filters on detection limits were evaluated.

The dried pressed pellet reference materials used in the WD-XRF measurement routine were placed on the stage of a Bruker Tracer portable ED-XRF spectrometer and the fluoresced XRF spectra captured using the Bruker S1PXF software program. Fluoresced X-rays were measured with a silicon drift detector for 120 s in air at 40 keV and 40 μA and used a removable multi-metal primary filter consisting of 25 μm Cu, 25 μm Ti, and 25 μm Al (Cu/Ti/Al filter). The average valid photon count rate was 16,815 photons per second with a resolution of 20.04 eV per channel at full height width for the Mn Kα 1 wavelength. The X-ray source of the spectrometer is a Rh-tube oriented at a 53-degree angle with respect to the sample. As the entire spectrum from 0 to 40 keV was captured by the software, routines were developed to quantify Cr, Mn, Ni, Cu, Zn, Pb, Y, and Cd. The captured spectra for the 10 μg g^−1^ reference material and the net Pb Lβ1 wavelength intensities for six reference materials are illustrated in Fig. 2. Calibration routines were developed by comparing net X-ray intensities to known dry weight element concentrations using the Bruker Microsoft Excel plugin (S1CalProcess). The peak overlap corrections, goodness of fit, root mean square error, calibration range, the LOD calculated using the background equivalent concentration approach (Thomsen, 2012) and the LOQ calculated (Thomsen et al., 2003) for each element are summarized in Table 2. The r^2^ of each calibration is no less than 0.97 with corresponding single-digit root mean square errors.

**Table 2:** Goodness of fit parameters for ED-XRF calibrations of pressed pellets with the Cu/Ti/Al primary filter. The Root Mean Square Error (RMSE) of the calibration, range in reference materials, Limit of Detection (LOD), and Limit of Quantitation (LOQ) are expressed in μg g^−1^, dry weight. The Kα1 wavelengths were used for each calibration, except for Pb which used the Lβ1 wavelength. Peak overlap corrections used in refining the calibration routines are summarized.

	Peak Overlap Corrections	Calibration r^2^	RMSE (μg g^−1^)	Range in Reference Materials (μg g^−1^)		LOD (μg g^−1^)	LOQ (μg g^−1^)
Cr	Fe Kα1	0.99	3.0	3.1	105	2.3	7.5
Mn	*None*	0.97	7.1	66.2	176	3.7	12.1
Ni	*None*	0.99	3.7	6.6	111	1.4	4.5
Cu	*None*	0.99	3.7	27.1	129	1.6	5.3
Zn	*None*	0.99	4.6	40.6	181	0.2	0.7
As	Pb Lα1	1.00	3.3	0.5	131	0.3	0.9
Pb	*None*	1.00	0.9	0.1	96	0.3	1.1
Y	*None*	0.99	17.8	0.5	600	1.5	4.9
Cd	*None*	0.94	11.2	0.6	119	0.2	0.6

To confirm that the infinite thickness observations made with WD-XRF remained valid for portable ED-XRF, the same reference materials were analyzed for 120 s and net intensities determined. A piece of 1 mm 99% pure lead foil was placed behind the pellet and the analysis repeated. The Pb foil was removed and a 0.025 mm piece of 99.9% pure Mo foil was placed behind the pellet and the analysis repeated a third time. The associated intensities increased with the presence of a metal foil, confirming the pressed pellets are infinitely thin with respect to the Pb Lβ1 and Mo Kα1 wavelengths.

Prior studies have counted fluoresced X-rays in plant material from 30 to 240 s (Bull et al., 2017, Gutiérrez-Ginés et al., 2013, Sacristan et al., 2016), or did not specify count times. To optimize the portable ED-XRF measurement routine and to quantify the improvement in the accuracy with an increase of time, four dried plant-based reference materials were analyzed by ED-XRF as described previously by varying measurement times from 5 s to 300 s. The resulting Pb concentrations were compared to the known Pb concentrations and the relative percent differences between each data pair calculated as described in Byers et al. (2016) (Fig. S2 of the Supplemental materials). The relative difference neared ± 10% at 120 s and only marginal improvements were noted with longer measurement time, therefore 120 s was selected as the default measurement time for the portable ED-XRF measurement routine.

Primary multi-metal filters have been designed by ED-XRF manufacturers to reduce variability in the energy of source X-ray photons and reduce background radiation. As illustrated in Fig. 2A, the Pb Lβ1 peak is located on the shoulder of the inelastic Compton radiation peak originating from the Rh tube. Normally, XRF measurements are normalized to this inelastic scatter (18.5–19.5 keV); however, normalization requires additional steps in the calibration. Therefore, the ED-XRF measurement and calibration steps outlined above were repeated using an alternative removable multi-metal primary filter consisting of 25 μm Ti, 50 μm Fe, and 25 μm Mo (Ti/Fe/Mo) designed to reduce the overall Compton radiation. The average valid photon count rate was 2931 photons per second with a resolution of 19.98 eV per channel at full height width for the Mn Kα1 wavelength. The peak overlap corrections, goodness of fit, root mean square error, calibration range, the LOD calculated using the background equivalent concentration approach (Thomsen, 2012) and the LOQ calculated (Thomsen et al., 2003) for each element summarized in Table S2 of Supplemental materials. Although the calibration goodness of fit parameters with the Ti/Fe/Mo multi-metal primary filter are reasonable (r^2^ values near one with single digits RMSE values), there is a small loss of fit in lower wavelengths.

#### 2.1.6 Confirmation of the dry plant-based portable ED-XRF measurement and calibration routine

The researchers grew common garden vegetables as described in Section 2.1.4 and samples prepared as described in Section 2.1.3. Each sample was analyzed by portable ED-XRF using the primary filter as described above. After measurement with the ED-XRF, 27 vegetable pressed pellets representing the range of measured lead concentrations were broken into 1 g aliquots and analyzed using ICP-MS as described in Section 2.1.3. The paired relationships between ICP-MS and ED-XRF measurements for Pb are illustrated in Fig. 3b.

To determine if changing primary multi-metal filters improved the applicability of the calibration to vegetable samples with respect to Pb, 35 additional samples of vegetables grown by the researchers were prepared as dried pressed pellets and analyzed using ED-XRF equipped with the Ti/Fe/Mo multi-metal primary filter. Concentrations were compared to measurements made using WD-XRF; the paired relationships between WD-XRF and ED-XRF measurements for Pb are illustrated in Fig. 3c. Although the calibrations for Pb using either primary filter are acceptable, the viability of the calibration routine to actual samples is better with the Cu/Ti/Al filter.

### 2.2 Wet plant-based ED-XRF measurement and calibration routines

#### 2.2.1 Preparation of wet plant-based reference materials

A major limitation to the use of portable ED-XRF in urban agriculture and food security applications is the lack of commercially available metals-rich raw (undried) plant-based reference materials. In developing undried reference materials analogous to raw vegetables, additional commercially sourced dried parsley was mixed with the liquid ICP metals standard and processed in a roto-vap as described previously to create a second library of eleven dried/homogenized parsley-based reference materials with Pb concentrations ranging from 0.5 μg g^−1^ to 100 μg g^−1^ on a dry-weight basis as confirmed by WD-XRF. The mean water content of vegetables grown in metals-rich soil used in this study is 87% with a standard deviation of 4%. To prepare a reference library analogous to raw vegetables, a 1.50 g aliquot of dried reference material was added to a glass vial containing 8.50 g of e-Pure water. The mixture was gently stirred, the jar lid tightly secured, and the mixture allowed to rest for 12 h. The process was repeated in a step-wise fashion to create a library of “wet” plant-based reference materials with Pb concentrations ranging from 0.0 μg g^−1^ to 15 μg g^−1^ wet weight. A blank reference was created using the same process. A second set of reference materials was created with 65% water for comparison purposes.

#### 2.2.2 Development of wet plant-based portable ED-XRF measurement and calibration routine

The 65% and 85% water content plant-based reference material sets were packed into single open-ended 32 mm diameter (10 ml volume) XRF sample cups (Premier Lab Supply; Port St. Lucie, FL) and secured with 4.0-µm polypropylene film (Premier Lab Supply). Each sample cup was analyzed via portable ED-XRF for 120 s using the Bruker S1PXF software program as described previously using the Cu/Ti/Al primary filter. The average valid photon count rates for reference materials with 65% and 85% water were 34,684 and 35,324 photons per second, respectively, with resolutions of 20.02 eV per channel at full height width for the Mn Kα wavelength. The measurement routine measured fluoresced X-rays for 120 s at 40 keV and 40 μA.

Calibration routines were developed to quantify Cr, Ni, Pb and Y. Custom calibration routines for each set of reference material were developed in Microsoft Excel using the Bruker plugin (S1CalProcess). The goodness of fit, root mean square error, calibration range, the LOD calculated using the background equivalent concentration approach (Thomsen, 2012) and the LOQ calculated (Thomsen et al., 2003) for each element is summarized on Table 3 for both water contents. The goodness of fit parameters for each element using the 85% water content reference set are less than those using the 65% water content reference set. Although the LOD and LOQ values for each element are similar between the two water contents, when converted from wet weight concentrations to dry weight concentrations, the calibration developed with 85% water content is weaker by comparison.

**Table 3:** Goodness of fit parameters for ED-XRF calibrations with the Cu/Ti/Al primary filter for plant-based reference materials consisting of 85 percent (%) and 65% water. The Root Mean Square Error of the calibration (RMSE), Limit of Detection (LOD), and Limit of Quantitation (LOQ) were measured in μg g^−1^ wet weight with ED-XRF, and these values converted to dry weight concentrations based on water content. The Kα1 wavelengths were used for each calibration, except for Pb which used the Lβ1 wavelength.

	Calibration r^2^	Concentration (μg g^−1^, wet weight)					Water Content	Concentration (μg g^−1^, dry weight)		
		RMSE	Range in Reference Materials		LOD	LOQ		RMSE	LOD	LOQ
Cr	0.70	3	0.3	17	3	11	85%	21	23	76
Ni	0.94	1	0.2	17	4	12		9	23	77
Pb	0.86	2	0.0	15	2	7		12	13	44
Y	0.90	10	0.2	96	1	2		66	4	14

Cr	0.97	2	1	41	4	13	65%	7	12	38
Ni	0.99	1	0.4	42	2	6		3	5	17
Pb	1.00	1	0.0	36	0.4	1		3	1	4
Y	0.95	17	0.4	234	4	14		48	12	41

#### 2.2.3 Confirmation of the wet ED-XRF measurement and calibration routine

Common garden vegetables were grown by the researchers in pots of metals rich soil sourced from residential vegetable gardens in the City of Milwaukee, Wisconsin. The vegetables were harvested, scrubbed vigorously under running water, and peeled (if necessary). Each vegetable tissue sample was coarsely homogenized in a food processor for 10 s. An aliquot of the homogenized slurry was poured into a 32 mm XRF sample cup and the sample cup secured with 4-µm polypropylene film. The sample cup was placed on the stage of a Bruker Tracer portable ED-XRF spectrometer and the fluoresced XRF spectra captured and analyzed as described in Section 2.2.2. The concentration of heavy metals was calculated based on the 85% water calibration routine. After analysis, the wet sample was dried and pressed pellets prepared as described in Section 2.1.2 and analyzed using the dried WD-XRF measurement routine.

To confirm the measurement depth of raw vegetables with portable ED-XRF, 67 vegetables grown in soil collected from residential vegetable gardens in Milwaukee, WI were coarsely homogenized, packed into sample holders, X-rays counted for 120 s, and net intensities determined. A piece of 1 mm 99% pure lead foil was placed behind the sample holder and the analysis repeated. The Pb foil was removed and a 0.025 mm piece of 99% pure Mo foil was placed behind the sample holder and the analysis repeated a third time. Based on the change in peak intensities, the samples are considered infinitely thick for the Pb Lβ1 wavelength, but not the Mo Kα1 wavelength.

### 2.3 Data evaluation

The r^2^ statistic is commonly used in bivariate calibration regressions to explain the amount of variability in the dependent variable (concentration) that can be explained by the independent variable (peak intensity). In XRF spectroscopy, researchers strive to maximize the r^2^ value as close to 1 as possible. Further, the XRF calibration routines are also evaluated with root mean square error (RMSE) values, which is the standard deviation of the calibration regression residuals. Most importantly, the RMSE can be interpreted in terms of measurement units of the dependent variable, which in this study is element concentration expressed in μg g^−1^. Therefore, in refining calibration routines, the r^2^ values were maximized and RMSE values minimized.

RMSE values were further used to evaluate the minimum element concentration quantifiable by each calibration. If the RMSE is less than the calculated LOQ, then the LOQ represents the smallest concentration that can be quantified by a calibration routine. However, if the RMSE is greater than the LOQ, then the RMSE represents the smallest concentration that can be quantified by the calibration routine. This approach allows for a greater certainty in the calibration and is more rigorous compared to the more common approach where the calibration range assumed to be represented by the range in concentration of calibration reference materials.

## 3 Results and discussion

### 3.1 Dried plant-based WD-XRF measurement and calibration routines

*Evaluation of measurement depth and the infinite thickness assumption.* Dried pressed pellets of vegetables of consistent mass considered infinitely thick with respect to CrKα1 and considered infinitely thin with respect to Pb Lβ1 were analyzed and the measurement depths confirmed with metal foils as described previously. Net intensities of the Pb Lβ1 wavelength increased 4 orders of magnitude when the lead foil was placed behind samples, thus confirming the pressed pellet samples are infinitely thin at 12.614 keV. Similarly, the net intensities of the Cu Kα1 fluoresced X-rays increased one order of magnitude confirming samples remained infinitely thin at 8.046 keV. When the “nickel-silver” foil was added behind the sample, the mean Ni Kα1 net intensity increased by 11 counts per second, which is a statistically significant increase (t < 0.001; 16 df) and equal to an increase of 77 μg g^−1^. The mean net intensity of the Mn Kα1 wavelength increased by 0.05 counts per second when the stainless steel foil was added, which is a statistically significant increase based on a matched-pair analysis (t < 0.01; 15 df), although the mean increase is equal to an increase of only 1 μg g^−1^. The Cr Kα1 (5.415 keV) net intensities and corresponding concentrations did not increase (t > 0.5; 15 df) when the stainless steel foil was added behind samples. This empirical evaluation matches the calculations of infinite thickness illustrated on Fig. 1, and by controlling for matrix effects, confirms that the dried vegetable pressed pellets used in this study are appropriate for use in measuring elements with fluoresced wavelengths less than 5.451 keV. Potential enhancement from absorption of characteristic X-rays generated in the housing is controlled.

Researchers are cautioned to evaluate multiple thicknesses of sample to quantify the impact thickness will have on measurement results for a particular element of interest. For example, although the concentration of Mn increased by 1 μg g^−1^ by adding foil behind the pellet, this increase may be within acceptable bias tolerances in some applications, especially if the target element is not present in the shielding and housing of the spectrometer or not subject to influence from absorption/enhancement by matrix elements.

*Evaluation of the dry plant-based WD-XRF measurement and calibration routine for Pb.* Measurements indicate Pb is not present in the housing of the WD-XRF, therefore, although the pellets are infinitely thin for the Pb Lβ1 wavelength, the housing of the WD-XRF is not a direct source of bias in these measurements. The RMSE value of the Pb WD-XRF calibration routine is greater than the LOD, but less than the LOQ; therefore, the calibration range for Pb varies between 1 and 96 μg g^−1^ (Table 1). Although RMSE and LOQ values from calibrations developed by others are not widely available in the literature, the LOD value for Pb in this study is one or more orders of magnitude less than values reported previously (Andersen et al., 2013, Gallardo et al., 2016, Gutiérrez-Ginés et al., 2013), and more importantly, the LOD (0.3 μg g^−1^) is equal to the Maximum Level for lead in leafy vegetables (on a dry weight basis) and only 0.1 μg g^−1^ greater than the Maximum Level for lead in cereal (WHO, 2018).

The appropriateness of the WD-XRF calibration routine for Pb developed in this study to actual vegetable samples was further confirmed with paired ICP-MS measurements. The slope of the bivariate regression between 23 WD-XRF and ICP-MS measurements of vegetables grown in metals-rich soil is 1.08 with an r^2^ value of 0.96 providing additional support in the accuracy of WD-XRF measurements (Fig. 3a).

*Evaluation of the dry plant-based WD-XRF measurement and calibration routine for Cr, Ni, and Y.* ICP-MS measurements confirmed the presence of Cr and Ni in the parsley used in this study. However, as RMSE values are less than LOQs and the XRF software calculated LODs are less than the Cr and Ni concentrations in the blank, the Cr and Ni calibrations are considered satisfactory for the element concentrations represented by the reference materials (Table 1). Although RMSE and LOQ values for calibrations are largely absent from the literature, our LOD values for these elements are less that previously achieved (Ni; (Gutiérrez-Ginés et al., 2013); Cu; (Gutiérrez-Ginés et al., 2013, Otaka et al., 2014)).

The concentrations of Y were minor in the vegetable samples; therefore, our evaluation of the WD-XRF routine with ICP-MS was not possible. However, measurements indicate Y is not present in the housing of the WD-XRF, therefore, although the pellets are infinitely thin for the Y Kα1 wavelength, the housing of the WD-XRF is not a direct source of bias in these measurements. Unfortunately, the concentrations of Cr and Ni are greater than the WD-XRF LOQs for only eight confirmation samples; therefore, our evaluation of the WD-XRF routine with ICP-MS is limited. Potential bias from absorption/enhancement due to the matrix and further enhanced by XRF shielding containing mixed metals is critical when evaluating transition metals in a carbon matrix. For instance, because the pressed pellets are infinitely thick for Cr Kα1, the presence of Cr in the housing/shielding of the XRF is not considered a direct source of bias in measurements. However, fluoresced Fe Kα1 X-rays from the shielding could be absorbed by Cr in the matrix and enhance fluorescence of Cr Kα1 X-rays. In addition, measurable quantities of Ni are present in the WD-XRF housing/shielding and the pellets are considered infinitely thin for Ni; therefore, characteristic Ni Kα1 X-rays generated by the XRF housing could return to the detector and serve as a source of direct bias. Or, Ni Kα1 characteristic X-rays generated by the shielding could be absorbed by Fe in the sample matrix, which could in turn enhance the generation of Cr Kα1 wavelengths. Therefore, researchers cannot ignore increased matrix enhancement due to the influence of shielding. To mitigate this potential source of bias, the same shielding/housing must be used for all samples so that critical enhancement influences can be controlled during development of the calibration routine.

### 3.2 Dried plant-based ED-XRF measurement and calibration routines

*Evaluation of measurement depth and the infinite thickness assumption.* This study focuses on the most common heavy metals, therefore optimization of ED-XRF calibration focused primarily on the spectrum between Cr Kα1 (5.415 keV) and Pb Lβ1 (12.614 keV) with less interest in the Y Kα1 (14.958 keV) to Cd Kα1 (23.173 keV) range.

Measurement depths of the dried pellets with portable ED-XRF were evaluated with metal foils as described previously. Similar to WD-XRF measurements, the Pb Lβ1 and Mo Kα1 intensities measured by ED-XRF increased significantly with the presence of the associated metal foil. Therefore, the pressed pellets are infinitely thin with respect to the Pb Lβ1 and Mo Kα1.

*Evaluation of the dry plant-based ED-XRF measurement and calibration routine for Pb.* The RMSE, LOD, and LOQ values for the portable ED-XRF calibration for lead with the Cu/Ti/Al multi-metal primary filter (Table 2) are nearly identical to the values for the WD-XRF calibration routine. Even more promising is the calibration accuracy that was achieved with the portable ED-XRF even though measurement count times are 5 times less than WD-XRF. Further, the confirmation of ED-XRF measurements with 27 paired ICP-MS measurements suggests the ED-XRF calibration routine with the Cu/Ti/Al multi-metal primary filter is accurate (regression r^2^ = 0.97; slope = 1.02) with no apparent bias (Fig. 3b).

The variation between the measured concentrations and known concentrations using four reference materials decreases sharply when portable ED-XRF measurement times are increased, with very little difference in RD values in measurements lasting 120 s or longer (Fig. S2 of Supplemental materials). Therefore 120 s analysis time is considered sufficient.

To reduce the Compton peak, the calibration and verification process summarized above was repeated using the a Ti/Fe/Mo multi-metal primary filter. Although the valid photon count rates reduced significantly with the second primary filter, the RMSE, LOD, and LOQ values (Table S2 of Supplemental materials) did not increase significantly compared to the first filter. However, the confirmation of Pb ED-XRF measurements with 35 paired WD-XRF measurements of vegetable samples indicate the ED-XRF calibration routine with the Ti/Fe/Mo multi-metal primary filter is weak (regression r^2^ = 0.91; slope = 1.39) with an unexplainable positive bias (Fig. 3c). Although the Ti/Fe/Mo primary filter decreased background radiation, use of the Cu/Ti/Al primary filter is preferable for quantification of lead in vegetables.

*Evaluation of the dry plant-based ED-XRF measurement and calibration routine for additional heavy metals.* Although Pb is the primary metal of interest in this study, the ED-XRF captured a wide spectrum range allowing for quantification of several metals. The relatively small RMSE and LOQ values suggest these elements can be accurately measured with ED-XRF down to the single-digit μg g^−1^ range (Table 2).

### 3.3 Wet plant-based portable ED-XRF measurement and calibration routines

*Evaluation of measurement depth and the infinite thickness assumption.* Based on a matched-pair statistical analysis, adding Pb foil behind the sample cups did not increase the net intensity of the Pb Lβ1 wavelength (p < 0.08, DF = 67) suggesting the coarsely homogenized wet (raw) samples packed into sample cups are infinitely thick for wavelengths less than 12.614 keV. However, adding Mo foil behind the sample cups significantly increased the net intensities of the Mo Kα1 wavelength (p < 0.001; DF = 66) confirming that samples are infinitely thin for wavelengths greater than 17.480 keV.

The RMSE, LOD, and LOQ values on a wet weight basis from the 85% water calibration of Cr, Ni, Pb, and Y (Table 3) are similar to values from the dry pressed pellets indicating X-ray attenuation by the presence of water is minimal. However, when converted to a dry weight basis the portable ED-XRF RMSE, LOD, and LOQ values from the wet plant-based calibration routine are comparatively large. Although the water in the reference materials does not significantly attenuate fluoresced X-rays, the water present in the matrix dilutes the quantity of heavy metals per unit volume of sample to a point where ED-XRF measurements of wet vegetables are not useful from a regulatory perspective unless the element concentrations are sufficiently large. Very few vegetables grown in this study had heavy metal concentrations greater than LOQs, therefore evaluation of the 85% water ED-XRF calibrations with confirmation samples was not possible.

Increasing the sample analysis time is the most common way to increase the precision and accuracy of XRF measurements and lower XRF detection limits. A supplemental ED-XRF calibration was developed using the 85% water content reference set by increasing the count time from 120 s to 300 s; calibration details are summarized in Table S3 of Supplemental material. By increasing the measurement time, the relatively small RMSE values from the calibrations converted to dry-weight concentrations based on water content suggests Cr, Ni, and Pb can be accurately measured with ED-XRF down to the single-digit μg g^−1^ range in raw samples (Table 3S). Although we were unable to empirically measure the LOD and LOQ using 300 s measurement time, increasing sample analysis time by a factor of 4 should reduce the detection limit by a factor of 2. If this relationship holds true for raw vegetable samples, based on the LOD at 120 s (Table 3), we expect the LOD for Pb with 300 s measurement time to decrease to near 1 μg g^−1^ (wet weight), which would be equivalent to approximately 7 μg g^−1^ on a dry weight basis.

The mean water content of 261 vegetable samples used in this study is 87% with a standard deviation of 4%, therefore, using the 65% water content calibration would be inappropriate to quantify heavy metals in our raw vegetables. However, this work suggests that if the water content of food samples is not more than 65% (for example grains or cereals), portable ED-XRF spectrometry could detect Pb in raw samples at concentrations as low as 1 μg g^−1^ dry weight (Table 3) with 120 s count time. Further, this suggests that portable ED-XRF is a viable technology for use in element quantification in prepared foods with low water contents.

## 4 Conclusions

This work has shown that by managing matrix effects, XRF can be a useful tool in quantification of lead and other heavy metals in vegetables. In our case, practical limits existed on the preparation of samples of sufficient thickness to retain all source and characteristic X-rays. Therefore, in addition to measuring samples and reference materials under the same conditions (e.g. energy, current, filter, count time, atmosphere), the most critical factors we managed in developing measurement routines for quantification of heavy metals in plant tissues with WD-XRF and portable ED-XRF were:

1. Developing reference materials with commutability to samples and maintaining consistency with sample preparation/handling (e.g. drying time, milling time, sample mass),
2. Selecting proper wavelengths to eliminate peak overlaps and controlling for possible enhancement from within the matrix or from characteristic X-rays generated by the shielding/housing,
3. Analyzing samples for long enough to maximize accuracy and precision, and
4. Confirming the viability of new routines to actual samples through paired analysis of samples with another quantification technology to provide additional assurance in the measurement and calibration routines.

As we hypothesized, by addressing these critical factors, this study demonstrates that WD-XRF and portable ED-XRF can be used to accurately and rapidly quantify heavy metals in vegetable samples with limits of detection achieving regulatory thresholds. Although the most robust calibration was obtained with WD-XRF, this technology is limited to fixed laboratory-based instruments. Slight compromise in the precision and accuracy of measurements with portable ED-XRF is offset by the portability and ease of use of this technology outside of a traditional laboratory setting.

Quantification of heavy metals in wet coarsely-homogenized raw (undried) vegetable tissues was performed; however, RMSE and LODs on a dry-weight basis are strongly influenced by measurement time and water content which currently limits this technology. However, the technology is very promising for analysis of coarsely homogenized wet (raw) foodstuffs with lower water contents, such as grains and legumes and could easily be adopted for prepared foods.

## Declaration of interest

None.

## Funding

This work was supported by the Children’s Health Environmental CORE Center at the University of Wisconsin Milwaukee, which is funded through the National Institute of Environmental Health Sciences [Grant No. P30ES004184].
