# Supplementary material for: XRF techniques to quantify heavy metals in vegetables at low detection limits
Source: Food Chem X. 2018 Dec 15;1:100001. doi: 10.1016/j.fochx.2018.100001 (PMC6694857; doi:10.1016/j.fochx.2018.100001)
Supplement: Supplementary data 1 [file mmc1.docx]

Figure S1. Possible secondary or tertiary enhancement due to absorption of characteristic X-rays in mixtures of (I) Zn, Ni, Fe, Cr; (II) Cu, Co, MN; and (III) Br, As.

|  | I) Zn-Ni-Fe-Cr.  Characteristic Zn Kα1 X-rays could be absorbed by Ni which could enhance fluorescence of Ni Kα1 X-rays. Characteristic Ni Kα1 X-rays could be absorbed by Fe which could enhance fluorescence of Fe Kα1 X-rays. Characteristic Fe Kα1 X-rays could be absorbed by Cr which could enhance fluorescence of Cr Kα1 X-rays. |
| --- | --- |
|  | II) Cu-Co-Mn.  Characteristic Cu Kα1 X-rays could be absorbed by Co which could enhance fluorescence of Co Kα1 X-rays. Characteristic Co Kα1 X-rays could be absorbed by Mn which could enhance fluorescence of Mn Kα1 X-rays. |
|  | II) Br-As.  Characteristic Br Kα1 X-rays could be absorbed by As which could enhance fluorescence of As Kα1 X-rays. |

Figure S2. Relative percent difference between ED-XRF measurements (with a Cu/Ti/Al primary filter) of lead and known values for four reference materials. Positive values indicate the expected concentration is greater than the measured concentration.
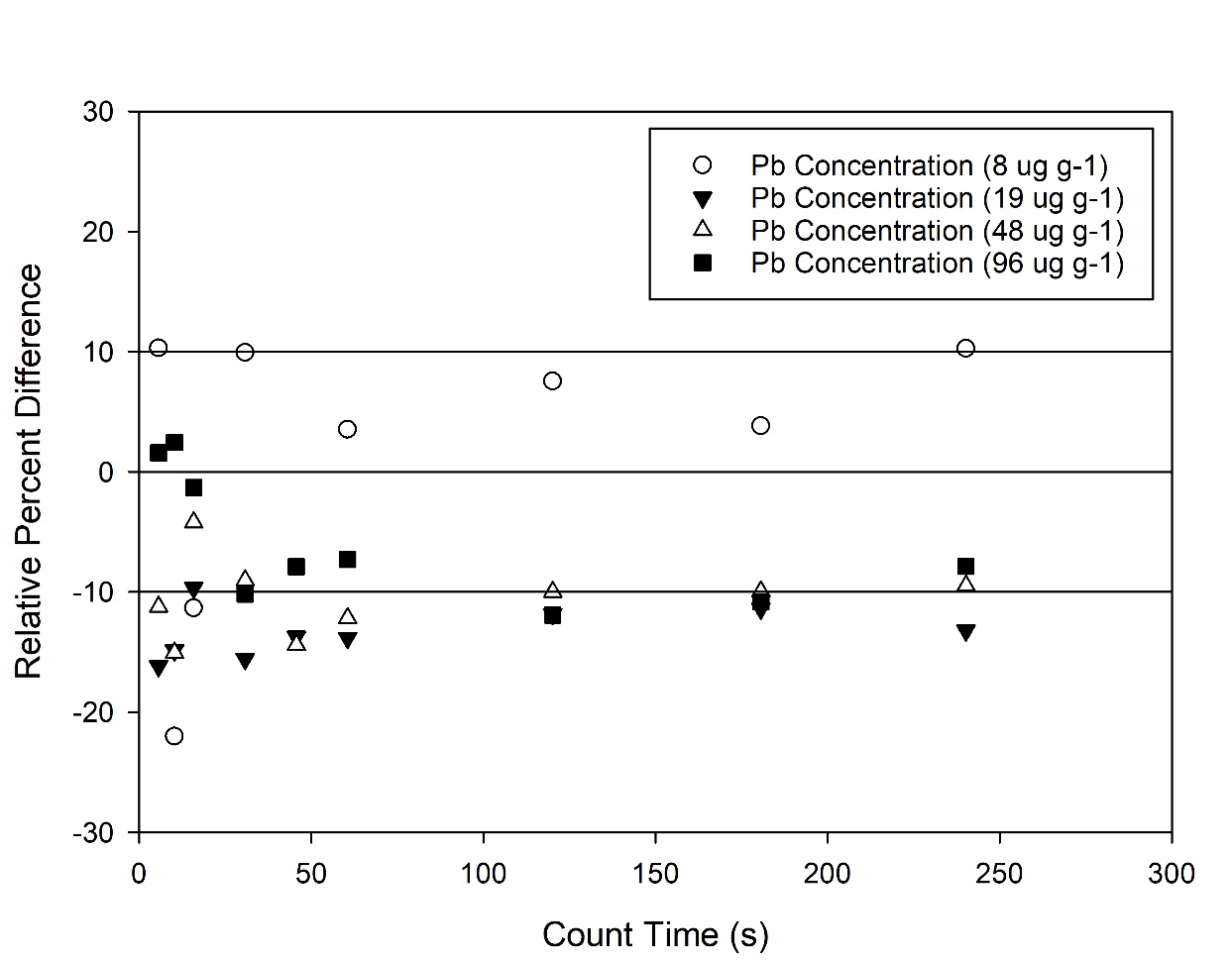


Table S1. WD-XRF measurement routine specifications.

Table S2. Goodness of fit parameters for ED-XRF calibrations with the Ti/Fe/Mo primary filter of pressed pellets. The Root Mean Square Error (RMSE) of the calibration, range in reference materials, Limit of Detection (LOD), and Limit of Quantitation (LOQ) are expressed in μg g-1, dry weight. The Kα1 wavelengths were used for each calibration, except for Pb which used the Lβ1 wavelength. Peak overlap corrections used in refining the calibration routines are included.

|  | Peak Overlap Corrections | Calibration r^2^ | RMSE  (μg g^-1^) | Calibration Range  (μg g^-1^) | | LOD  (μg g^-1^) | LOQ  (μg g^-1^) |
| --- | --- | --- | --- | --- | --- | --- | --- |
| Cr | Fe Kα1 | 0.84 | 15.6 | 3.1 | 105 | 0.9 | 3.0 |
| Mn | *none* | 0.88 | 14.4 | 66.2 | 176 | 5.8 | 19.0 |
| Ni | *none* | 1.00 | 1.5 | 6.6 | 111 | 3.3 | 10.8 |
| Cu | *none* | 0.93 | 9.9 | 27.1 | 129 | 1.4 | 4.6 |
| Zn | *none* | 0.96 | 9.7 | 40.6 | 181 | 1.2 | 4.0 |
| As | Pb Lα1 | 1.00 | 1.5 | 0.5 | 131 | 1.1 | 3.6 |
| Pb | *none* | 1.00 | 1.4 | 0.1 | 96 | 0.9 | 2.9 |
| Y | *none* | 1.00 | 0.8 | 0.0 | 600 | 0.1 | 0.4 |
| Cd | *none* | 0.99 | 3.9 | 0.6 | 119 | 10.0 | 32.9 |

Table S3. Goodness of fit parameters for ED-XRF calibrations with the Cu/Ti/Al primary filter for plant-based reference materials consisting of 85 percent (%) with 300 s count time. The Root Mean Square Error (RMSE) of the calibration was measured in μg g^-1^ (wet weight) and these values converted to dry weight based on water content. The Kα1 wavelengths were used for each calibration, except for Pb which used the Lβ1 wavelength.

|  | Calibration r^2^ | Concentration (μg g^-1^, wet weight) | | | Water Content | Concentration (μg g^-1^, dry weight) |
| --- | --- | --- | --- | --- | --- | --- |
|  |  | RMSE | Range in Reference Materials | |  | RMSE |
| Cr | 0.96 | 1.1 | 0.3 | 17 | 85% | 7 |
| Ni | 0.99 | 0.4 | 0.2 | 17 |  | 2.5 |
| Pb | 0.99 | 0.2 | 0.0 | 15 |  | 1.1 |
| Y | 0.99 | 3.8 | 0.2 | 96 |  | 25 |
